# Machine-learning–guided transcriptomic integration identifies GFM1 as a lactylation-related candidate biomarker in aortic dissection

**DOI:** 10.1038/s41598-026-40139-9

**Published:** 2026-02-14

**Authors:** Junquan Chen, Nan Jiang, Zhigang Guo, Yunpeng Bai

**Affiliations:** 1 Department of Cardiovascular Surgery, Chest Hospital, Tianjin University, Tianjin, 300222 China; 2 Tianjin Medical University, Tianjin, 300070 China

**Keywords:** Aortic dissection, Lactylation, Integrative transcriptomics, Machine learning, GFM1, Vascular smooth muscle cells, Biomarkers, Cardiology, Computational biology and bioinformatics, Diseases, Medical research

## Abstract

Aortic dissection (AD) is a life-threatening aortic disease with limited disease-modifying pharmacologic options. Lysine lactylation is a metabolism-linked post-translational modification implicated in vascular and inflammatory biology, but its relationship to AD has not been well characterized. Public AD transcriptomic datasets were integrated for differential expression analysis and WGCNA. Lactylation-related DEGs were defined by intersecting DEGs with a curated lactylation-related gene set. Candidate genes were prioritized using complementary machine-learning models (LASSO, Random Forest, and XGBoost) as a feature-screening strategy with internal resampling and hold-out validation (cross-validation and a hold-out set). GFM1 expression was assessed by qRT-PCR and western blotting in human aortic tissues. Functional relevance was examined in primary mouse aortic vascular smooth muscle cells (VSMCs) using siRNA knockdown under angiotensin II stimulation (1.0 µmol/L, 24 h), with proliferation and migration assessed by CCK-8, EdU, Transwell, and scratch-wound assays. We identified 217 DEGs and an AD-associated co-expression module. Intersection analysis yielded 11 lactylation-related DEGs, among which GFM1 received consistent support across models. GFM1 showed higher expression in AD tissues, and GFM1 knockdown attenuated angiotensin II–induced VSMCs proliferation and migration. Integrated transcriptomics and machine-learning–based prioritization nominate GFM1 as a lactylation-related candidate associated with AD, warranting further investigation. These findings are hypothesis-generating: model performance reflects internal evaluation only, and independent external validation and direct lactylation profiling are required to establish generalizability and clarify mechanistic links.

**Supplementary Information:**

The online version contains supplementary material available at 10.1038/s41598-026-40139-9.

## Introduction

 Aortic dissection (AD) is a catastrophic cardiovascular condition with abrupt onset, rapid progression, and high mortality^1^. Pathologically, an intimal tear permits blood to enter the media, creating an intramural hematoma and propagating the separation of the intima from the media. The annual incidence is ~ 1 per 100,000, with a higher prevalence in men than in women^2^. Without timely intervention, many patients die within hours to days of symptom onset, and mortality is estimated to increase by 1%–2% per hour during the first 24 hours^3^. Despite advances in perioperative care, no approved disease-modifying pharmacologic therapy exists for AD, surgery/endovascular repair remains the mainstay for many patients^4^. However, substantial perioperative risk and postoperative complications can compromise survival and quality of life, while prolonged intensive care and hospitalization impose considerable economic burdens on patients and society^5–8^. These challenges underscore the need to identify reliable biomarkers and actionable targets and to generate testable mechanistic hypotheses for future translational studies.

Lysine lactylation is a recently described post-translational modification (PTM) in which lactate-derived acyl groups can be added to lysine residues on proteins^9^. Unlike many classical PTMs, lactylation has been discussed as a potential link between cellular metabolic state and gene regulation, with reported roles in immune modulation, epigenetic remodeling, and stress adaptation^10,11^. In cancer, for example, lactylation has been implicated in shaping the tumor microenvironment and influencing proliferation, migration, and survival^12^. By contrast, its relevance to vascular pathologies—particularly AD—remains poorly defined.

The pathogenesis of AD involves vascular smooth muscle cells (VSMCs) dysfunction, inflammatory activation, and structural remodeling of the aortic wall^13,14^. In this study, the term “lactylation-related genes” refers to genes curated from prior literature/databases that are implicated in lactate metabolism and/or lactylation-associated regulatory programs; it does not necessarily indicate that the corresponding proteins are directly lysine-lactylated in AD. Given lactylation’s regulatory roles in immune and metabolic pathways, it is plausible that lactylation-related genes (LRGs) contribute to AD initiation and progression; however, systematic investigations in AD are scarce. Bioinformatics has become integral to discovering disease biomarkers and dissecting molecular networks in complex conditions^15,16^. Integrating bioinformatics with machine learning to delineate disease subtypes and prioritize candidate markers represents a powerful interdisciplinary strategy^17^. Importantly, our goal here is candidate nomination and hypothesis generation within a lactylation-related transcriptomic framework, rather than lactylation-level mechanistic proof.

In this study, we integrated public transcriptomic datasets with a machine-learning prioritization pipeline to examine lactylation-related transcriptional signatures in AD. By screening a curated lactylation-related gene set within AD expression profiles, we prioritized GFM1 as a candidate associated with AD and conducted preliminary tissue-level and cellular validation. These results provide hypothesis-generating insights and a rationale for subsequent studies, including validation in independent cohorts and direct lactylation profiling, to establish robustness and mechanism.

## Methods

### Public data download and processing

**Fig. 1:**
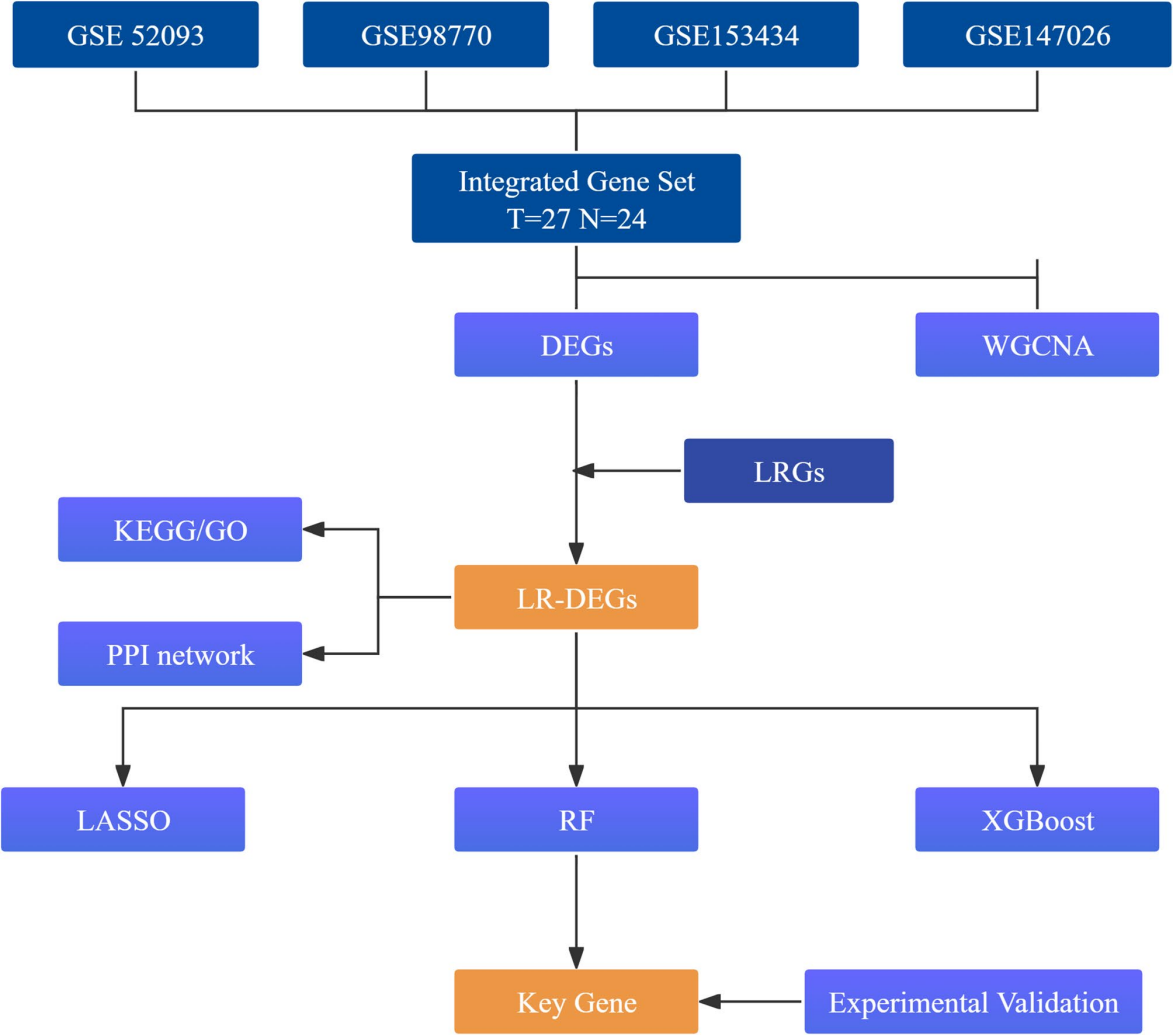
Study design workflow. Integrated transcriptomic profiles of AD and control aortas were analyzed by differential expression and WGCNA to identify disease-associated genes/modules. Lactylation-related DEGs were defined by intersecting DEGs with a curated lactylation-related gene set, followed by enrichment and PPI analyses. Key candidates were prioritized by machine learning and validated experimentally.

The overall workflow is shown in Fig. 1. Public AD transcriptomic datasets (GSE52093, GSE98770, GSE153434, GSE147026) were downloaded and preprocessed in R (v4.2.1). For microarray datasets, background correction and between-array normalization were performed using limma’s normalizeBetweenArrays^18^. Expression values were log2-transformed when required (i.e., when data were not already on a log2 scale) to improve comparability while preserving relative differences. After mapping probes to official gene symbols and harmonizing identifiers across platforms, the four datasets were merged, yielding a combined matrix of 27 AD and 24 control aortic samples. Batch effects were adjusted using ComBat (sva package) with study/source as the batch variable^19^, with AD status included as a covariate in the model matrix to preserve biological signal. Principal component analysis (PCA) before and after ComBat indicated substantial mitigation of batch effects while preserving group-related variability, providing a basis for downstream analyses.

### Identification of differentially expressed genes

Differential expression was assessed using limma with empirical Bayes moderation. Genes were considered significant at |log2FC| > 0.26 (≈ 1.2-fold) and FDR < 0.05 (Benjamini–Hochberg). Genes with positive log2FC were defined as upregulated in AD, and those with negative log2FC as downregulated. Results were visualized using volcano plots (ggplot2) and heatmaps (pheatmap).

### Co-expression network construction via WGCNA

Genes were variance-filtered (top 60% by variance) prior to WGCNA^20^. The soft-thresholding power was selected to approximate scale-free topology (scale-free topology fit index R² ≈ 0.8). A signed network was constructed with power = 8, minModuleSize = 30, and mergeCutHeight = 0.25, followed by topological overlap computation and module detection. Module–trait correlations were calculated, and the module showing the strongest association (highest absolute correlation) with AD status was prioritized for downstream analyses.

### Acquisition of lactylation-related differentially expressed genes

**Fig. 2:**
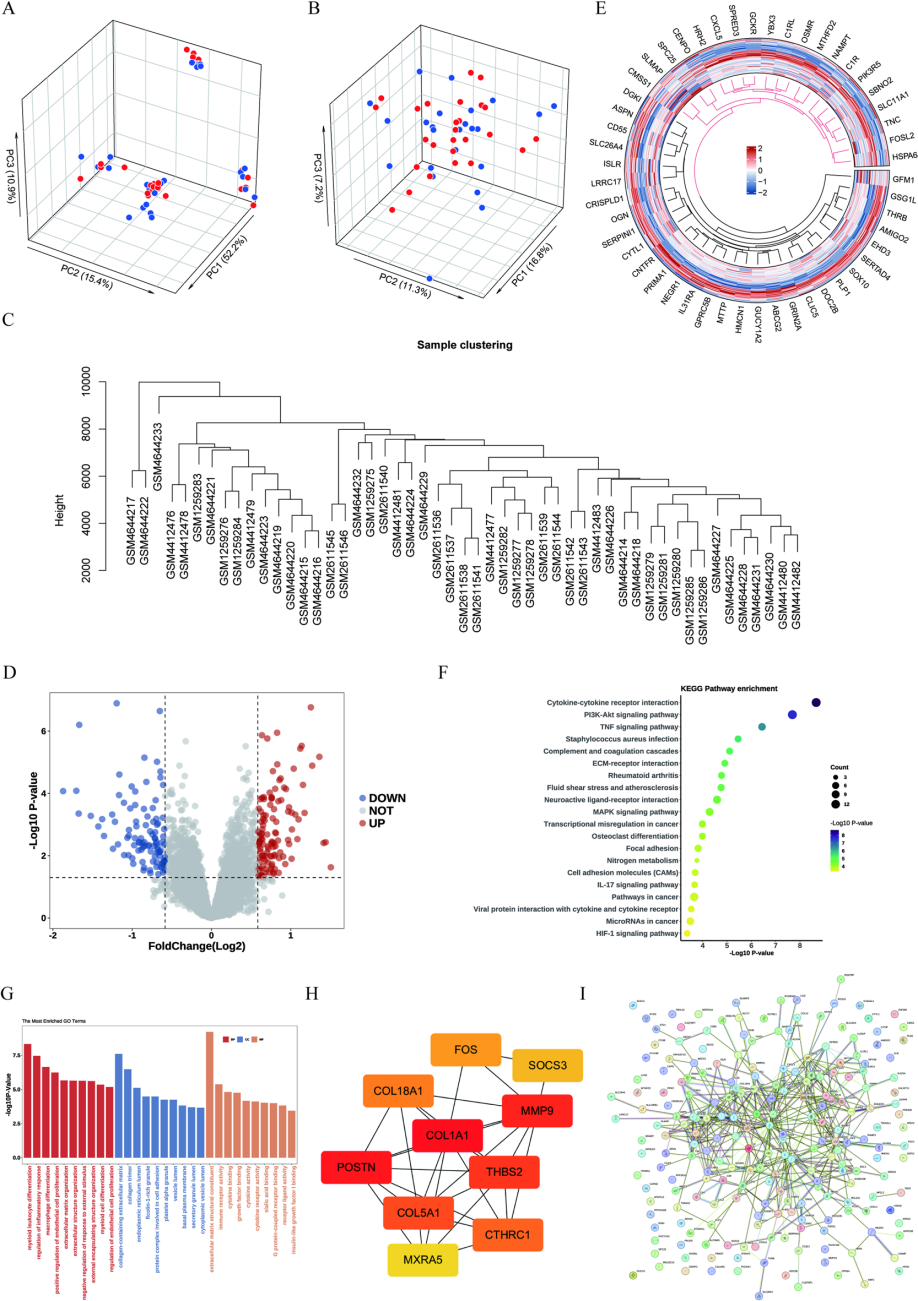
Transcriptomic differences between AD and controls and functional enrichment. (**A**) 3D PCA before batch correction; (**B**) 3D PCA after batch correction (blue, controls, *n* = 24; red, AD, *n* = 27). (**C**) Hierarchical clustering of samples. (**D**) Volcano plot of DEGs. (**E**) Circular heatmap of the top 50 DEGs (row Z-scores). (**F**) KEGG enrichment bubble plot (x-axis and color: −log10(P value); size: Count). (**G**) GO enrichment bar plot (y-axis: −log10(P value)) across BP/CC/MF. (**H**) Hub-gene interaction subnetwork. (**I**) Global PPI network of DEGs. Enrichment significance was assessed using Benjamini–Hochberg adjusted P values (p.adjust < 0.05), whereas plots display −log10(P value) for visualization. KEGG: Kyoto Encyclopedia of Genes and Genomes (see References^23^ and^24^).

A lactylation-related gene set was curated from public resources by querying lactylation- and lactate metabolism–related programs in the Molecular Signatures Database (MSigDB) gene set collections^21^. Intersecting this gene set with differentially expressed genes (DEGs) yielded lactylation-related DEGs (LR-DEGs), and overlaps were displayed using Venn diagrams. Importantly, this gene-set restriction provides a transcriptomic framework and does not constitute direct quantification of protein or histone lactylation. STRING (confidence > 0.75) was used to derive protein–protein interaction networks, and clusterProfiler^22^was used for GO/KEGG^23,24^ enrichment analysis with p.adjust < 0.05. For visualization, Fig. 2F labels −log10(P value) as shown, whereas statistical significance was determined using adjusted P values (p.adjust) with a cutoff of 0.05.

### Identification of the key gene

Samples were randomly split into stratified training and validation cohorts (75%/25%) with a fixed random seed (seed = 2024) for reproducibility. Model tuning was performed within the training set using 5-fold cross-validation. LASSO logistic regression was implemented using glmnet (family = binomial, alpha = 1), and candidate genes were defined as those with non-zero coefficients at the λ1se solution (1-SE rule). Random Forest (ntree = 1000) and XGBoost models were trained on the same training set, and feature importance was extracted from each model. Model performance was summarized as internal AUC on the hold-out validation set; no independent external cohort was used for validation in this study. Cross-model consensus was defined a priori using a vote-based overlap criterion across LASSO, Random Forest, and XGBoost: one vote if a gene was selected by LASSO (non-zero coefficient at λ1se), and one vote each if it ranked among the top three (K = 3) features by importance in Random Forest and XGBoost. Genes with a consensus score of 3/3 were prioritized for downstream validation.

### Function enrichment analysis

Complementary pathway interrogation was performed using KOBAS 3.0^25^ and clusterProfiler. Enrichment results were visualized using dotplots, in which the x-axis (and color scale) represents −log10(P value). Statistical significance was determined using Benjamini–Hochberg adjusted P values (p.adjust < 0.05). Results were plotted with ggplot2.

### Gene set enrichment analysis

GSEA was conducted in clusterProfiler using the MSigDB C2: CP KEGG collection (c2.cp.kegg.v2022.1.Hs.symbols.gmt), reporting NES, nominal P, and FDR q-values; significance was defined as FDR < 0.25. Plots were generated with GseaVis.

### Protein-protein interaction analysis

STRING-derived networks were complemented with GeneMANIA (default settings) to construct broader interaction maps and highlight putative functional neighborhoods.

### Quantitative reverse transcription PCR (qRT-PCR)

Human ascending aortic tissues (AD vs. control) were obtained from Tianjin Chest Hospital after written informed consent and were approved by the Ethics Committee of Tianjin Chest Hospital (Approval No. 2023LW-001). All methods were performed in accordance with the relevant guidelines and regulations and with the Declaration of Helsinki. Human aortic tissues were then processed for RNA extraction and cDNA synthesis. qRT-PCR was performed in technical triplicates using β-actin as the internal reference. Relative expression was calculated by the 2^−ΔΔCt^ method. The sample size for each group is reported in the corresponding figure legends. Primer sequences were as follows:

**Table:** 

Gene name	Forward	Reverse
GFM1	ATGGTGAGGTTGGCATGAGA	TGGGCTGATGAGTCTCTGTT
β-Actin	AGAGCTACGAGCTGCCTGAC	AGCACTGTGTTGGCGTACAG

### Western blotting of human aortic tissues

Protein was extracted from snap-frozen human aortic tissues (AD and control) using RIPA buffer with protease/phosphatase inhibitors. Equal amounts of protein were separated by SDS-PAGE and transferred to PVDF membranes. After blocking (5% BSA), membranes were incubated with primary antibodies against GFM1 and β-ACTIN, followed by HRP-conjugated secondary antibodies. Bands were visualized by enhanced chemiluminescence and quantified by ImageJ; GFM1 intensity was normalized to β-ACTIN. Group sizes and replicate numbers are reported in figure legends.

### Mouse aortic VSMCs culture, SiRNA transfection targeting GFM1, and functional assays

Mouse aortic VSMCs were cultured in Dulbecco’s modified Eagle’s medium supplemented with 10% fetal bovine serum and 1% penicillin/streptomycin at 37 °C in 5% CO_2_. Cells were transfected with siRNA targeting GFM1 (siGFM1) or negative-control siRNA (siNC) using a lipid-based transfection reagent according to the manufacturer’s instructions (final siRNA concentration: 50 nM). After recovery, cells were stimulated with angiotensin II (AngII, 1.0 µmol/L for 24 h) or vehicle as indicated. Four experimental conditions were used: Control (untreated), Model (AngII only), NC (siNC + AngII), and siGFM1 (siGFM1 + AngII).

*SiRNA knockdown efficiency*: Knockdown efficiency was quantified 24 h after transfection by qRT-PCR (2^^−ΔΔCt^, normalized to β-actin). Quantified knockdown data are provided in Supplementary Figure S1.

*CCK-8*: Cell viability/relative proliferation was assessed using the CCK-8 kit. For each independent experiment, each condition was plated in technical triplicates (three wells). Cells were exposed to AngII (or vehicle) as specified, and OD450 was recorded at 24 h after AngII treatment. Technical replicates were averaged to yield one value per biological replicate.

*EdU*: DNA synthesis was measured using an EdU incorporation assay. Cells were incubated with EdU for 2 h, followed by fixation and nuclear counterstaining. For each condition, EdU-positive nuclei were quantified as a percentage of total nuclei from ≥ 5 random fields per well under identical imaging settings.

*Transwell migration*: Serum-starved cells were seeded into the upper chamber of 8-µm-pore inserts in serum-free medium, with 10% FBS in the lower chamber as chemoattractant. After 24 h, migrated cells were fixed, stained with crystal violet, and counted in ≥ 5 non-overlapping fields per insert.

*Scratch (wound-healing)*: Confluent monolayers were scratched using a 200-µL pipette tip, rinsed to remove debris, and cultured in 1% FBS medium with AngII or vehicle as indicated. Images were captured at 0 h and 12 h under identical settings; wound area was quantified in ImageJ and expressed as percent closure relative to 0 h.

### Statistical analysis

Analyses were performed in R (v4.2.1) and GraphPad Prism (v9). Data are reported as mean ± SD or median (IQR) and compared using unpaired t tests or Mann–Whitney U tests (two groups), and one-way ANOVA with Tukey’s post hoc or Kruskal–Wallis with Dunn’s post hoc (≥ 3 groups); categorical data were compared using χ² or Fisher’s exact tests. For cell assays, statistics were conducted at the biological-replicate level. Two-sided *P* < 0.05 was considered significant. Transcriptome-wide analyses controlled multiplicity using Benjamini–Hochberg FDR. For cell-based assays, biological replicates refer to independent experiments performed on different days (*n* = 3), whereas technical replicates (e.g., multiple wells per condition and multiple imaged fields per well/insert) were averaged within each experiment before statistical testing.

## Results

### Characterization of differentially expressed genes and functional associations in AD

PCA showed separation between AD and control samples after batch-effect adjustment, consistent with improved comparability in the integrated dataset (Fig. 2A–B). Unsupervised hierarchical clustering broadly recapitulated the group structure (Fig. 2C). Comparative transcriptomic analysis identified 217 DEGs (117 upregulated and 100 downregulated) at the prespecified thresholds in AD versus controls (Fig. 2D). A radial heatmap depicts expression patterns of the top 50 DEGs (Fig. 2E). Enrichment analyses suggested involvement of immune/inflammatory and extracellular-matrix remodeling pathways. KEGG enrichment highlighted cytokine–cytokine receptor interaction, PI3K–Akt signaling, and TNF signaling (Fig. 2F), while Gene Ontology terms were predominantly related to myeloid leukocyte differentiation and extracellular matrix organization (Fig. 2G). In the protein–protein interaction network, COL1A1, MMP9, and POSTN showed relatively high connectivity, consistent with central roles within the inferred network (Fig. 2H–I).

### Co-expression network architecture and disease-relevant module detection via WGCNA

**Fig. 3:**
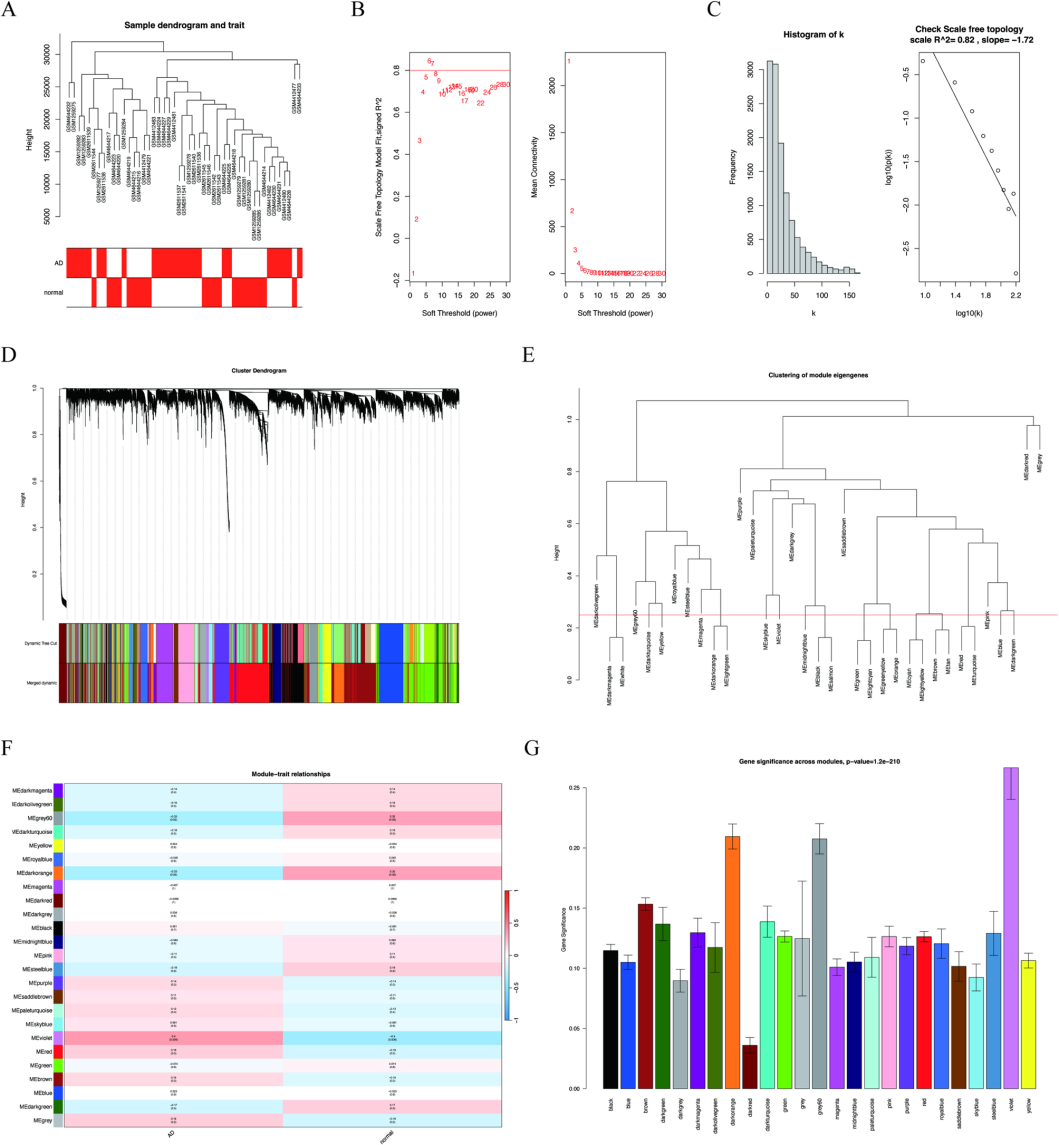
WGCNA network construction and identification of AD-associated modules. (**A**) Sample clustering dendrogram of the expression profiles used for WGCNA. The trait heatmap indicates disease status (AD vs. control), where red denotes higher trait values and white denotes lower trait values. (**B**) Soft-threshold power (β) selection: scale-free topology fit index (left) and mean connectivity (right) across candidate β values. The horizontal line indicates the target scale-free fit; β = 8 was selected for network construction. (**C**) Network connectivity distribution (k; left) and scale-free topology check (log10(k) versus log10(p(k)); right). (**D**) Gene dendrogram and module detection. Color bars show modules identified by dynamic tree cut and modules after merging. (**E**) Clustering of module eigengenes; modules were merged at mergeCutHeight = 0.25 (eigengene correlation > 0.75). (**F**) Module–trait relationship heatmap showing Pearson correlation coefficients and corresponding P values. (**G**) Summary of gene significance across modules.

WGCNA identified co-expression modules associated with AD. Sample clustering with trait annotation is shown in Fig. 3A. Using a scale-free topology framework with a soft-threshold power of 8, the network achieved a scale-free topology fit index of R² = 0.82 (slope = − 1.72; Fig. 3B–C). Gene dendrogram and dynamic tree cut identified 25 modules (Fig. 3D). Modules were further merged based on eigengene similarity (mergeCutHeight = 0.25; Fig. 3E). Module–trait analysis showed that the MEviolet module exhibited the strongest association with AD status (*r* = 0.40; *p* = 0.006; Fig. 3F) and was prioritized for downstream analyses. Gene significance patterns across modules are summarized in Fig. 3G.

### Lactylation-related DEGs and pathway enrichment

**Fig. 4:**
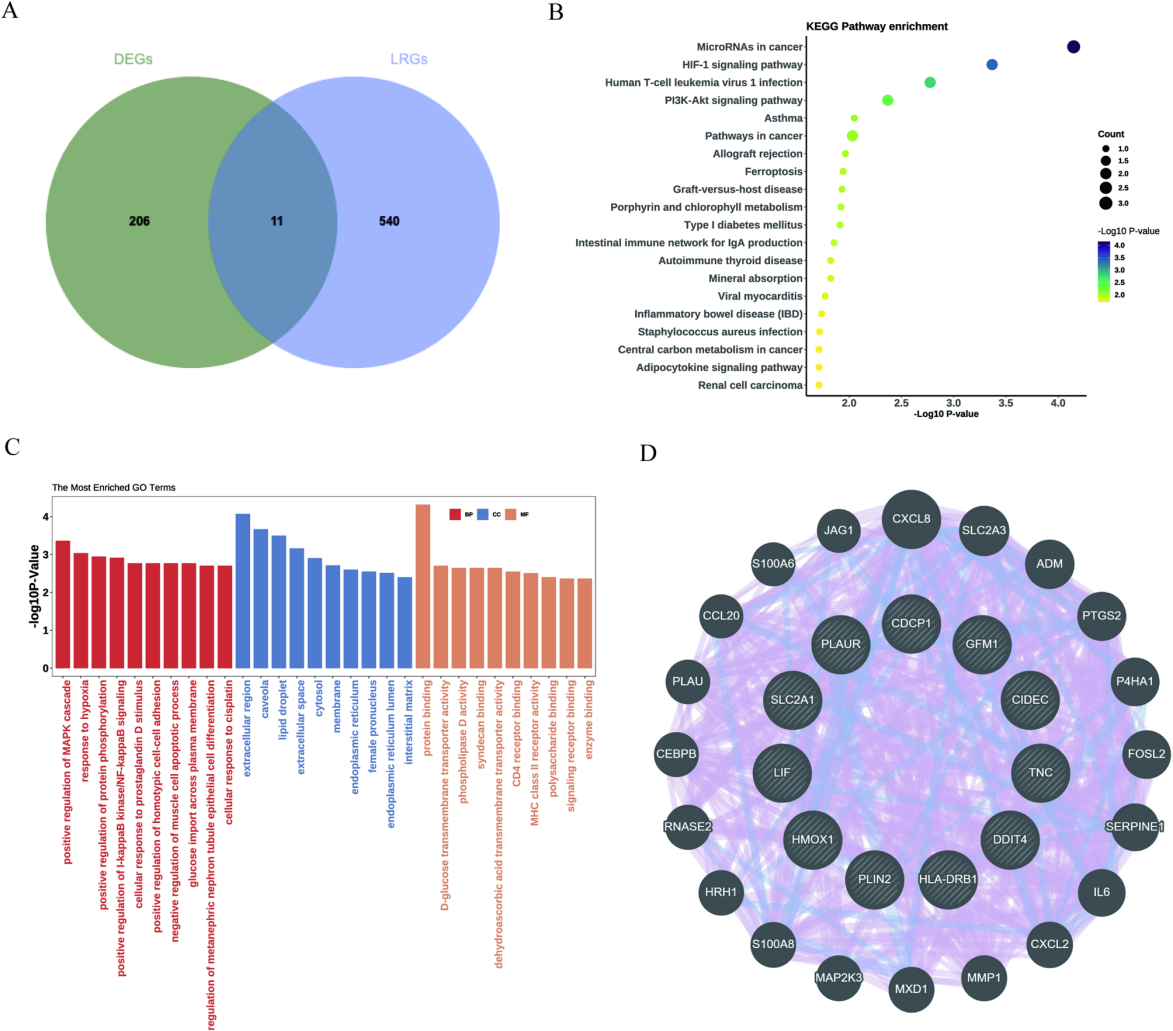
Identification and annotation of lactylation-related DEGs (LR-DEGs). (**A**) Venn diagram showing the overlap between DEGs and the lactylation-related gene set, yielding 11 LR-DEGs. (**B**) KEGG pathway enrichment of LR-DEGs displayed as a bubble plot; the x-axis indicates −log10(P value), bubble size denotes Count, and bubble color represents −log10(P value). (**C**) GO enrichment of LR-DEGs; bars are grouped by BP/CC/MF as indicated by colors, and the y-axis shows −log10(P value). (**D**) Protein–protein interaction (PPI) network of LR-DEGs; nodes represent genes and edges represent predicted interactions. Enrichment significance was assessed using Benjamini–Hochberg adjusted P values (p.adjust < 0.05), whereas plots display −log10(P value) for visualization. KEGG: Kyoto Encyclopedia of Genes and Genomes (see References^23^ and^24^).

Intersecting DEGs with a curated lactylation-related gene set identified eleven LR-DEGs (Fig. 4A). Functional annotation suggested enrichment of hypoxia-responsive signaling (including the HIF-1 pathway) as well as immune- and metabolism-related processes (Fig. 4B). Gene Ontology mapping highlighted regulation of the MAPK cascade, extracellular localization, and protein domain interactions (Fig. 4C). Within the LR-DEG protein–protein interaction network, GFM1, CIDEC, and DDIT4 exhibited relatively higher degree centrality, indicating that these genes may occupy more connected positions within the inferred network (Fig. 4D).

### Identification and validation of key genes using LASSO, random Forest, and XGBoost algorithms

**Fig. 5:**
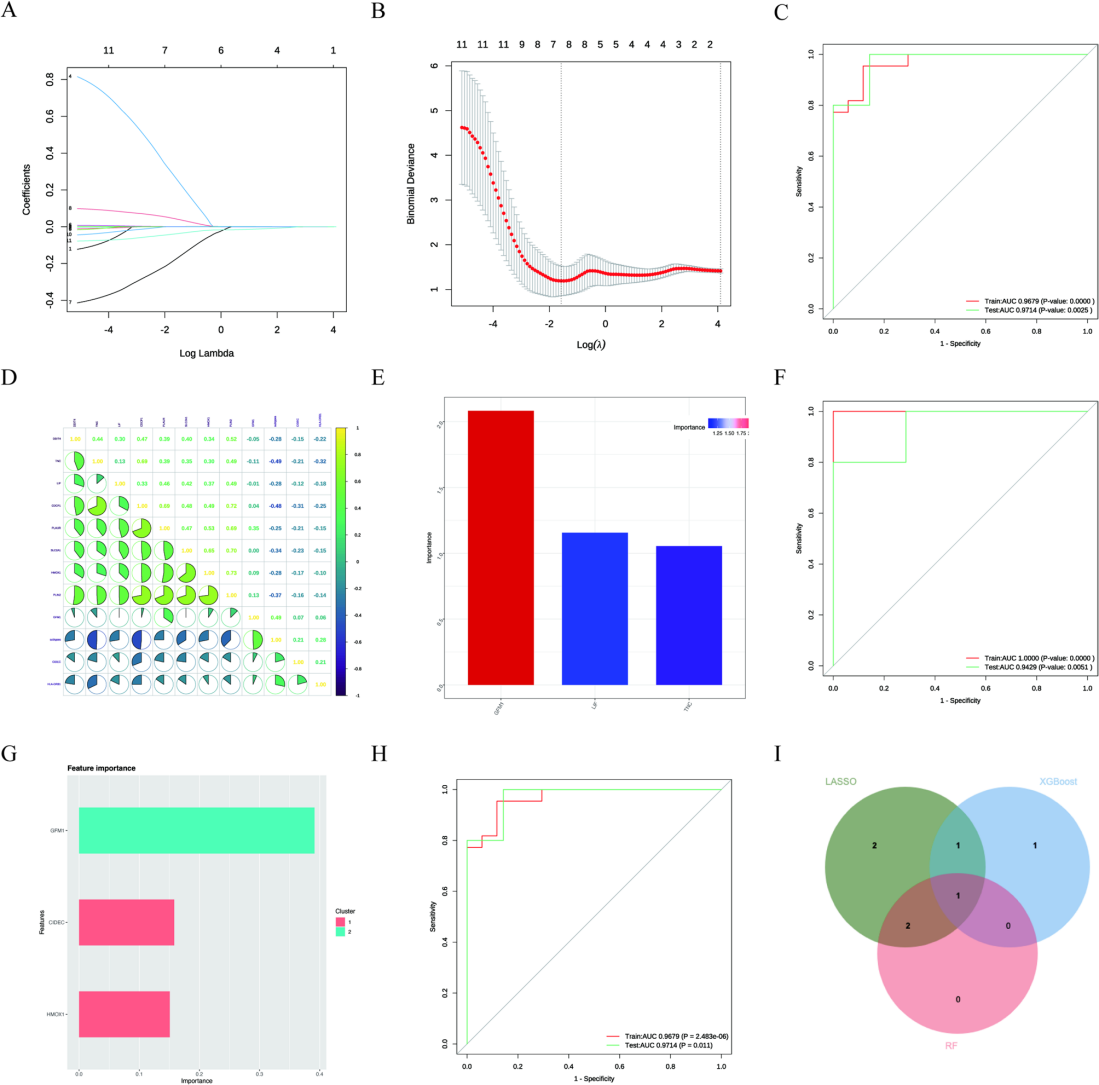
Machine-learning prioritization and model performance. (**A**) LASSO coefficient profiles across log(λ). (**B**) Cross-validation curve for LASSO; the two vertical dotted lines indicate λmin and λ1se, and λ was selected using the 1-SE rule. (**C**) ROC curves for the LASSO model (train/test curves with AUC values shown in the panel). (**D**) Correlation matrix of candidate genes; color indicates correlation direction and magnitude (|r|). (**E**) Random Forest feature-importance ranking (top features shown). (**F**) ROC curves for the Random Forest model (train/test metrics shown in the panel). (**G**) XGBoost feature-importance ranking; features are grouped into clusters (e.g., Cluster 1/2) as labeled. (**H**) ROC curves for the XGBoost model (train/test metrics shown in the panel). (**I**) Venn diagram showing the overlap of model-specific candidate sets; “cross-model consensus” was defined as genes supported by all three models (LASSO-selected genes at λ1se and the top three features by importance in Random Forest and XGBoost), and the intersection identifies the final key candidate (GFM1).

Using the eleven LR-DEGs, LASSO regression identified six candidates with non-zero coefficients at the λ1se solution: LIF, TNC, DDIT4, HMOX1, PLAUR, and GFM1 (Fig. 5A–D). Random Forest and XGBoost ranked genes based on feature-importance measures (Fig. 5E–H). We quantitatively defined “cross-model consensus” as a vote-based overlap across the three models: one vote for LASSO selection (non-zero coefficient at λ1se) and one vote each for ranking among the top three features by importance in Random Forest and XGBoost, yielding a consensus score of 0–3. The overlap is summarized in the Venn diagram (Fig. 5I). Under this pre-defined criterion, GFM1 was the only gene supported by all three models (score = 3/3) and was therefore prioritized for downstream validation. AUC values reflect internal discrimination only and should be interpreted cautiously in the absence of independent external validation.

### Validation of GFM1 expression

**Fig. 6:**
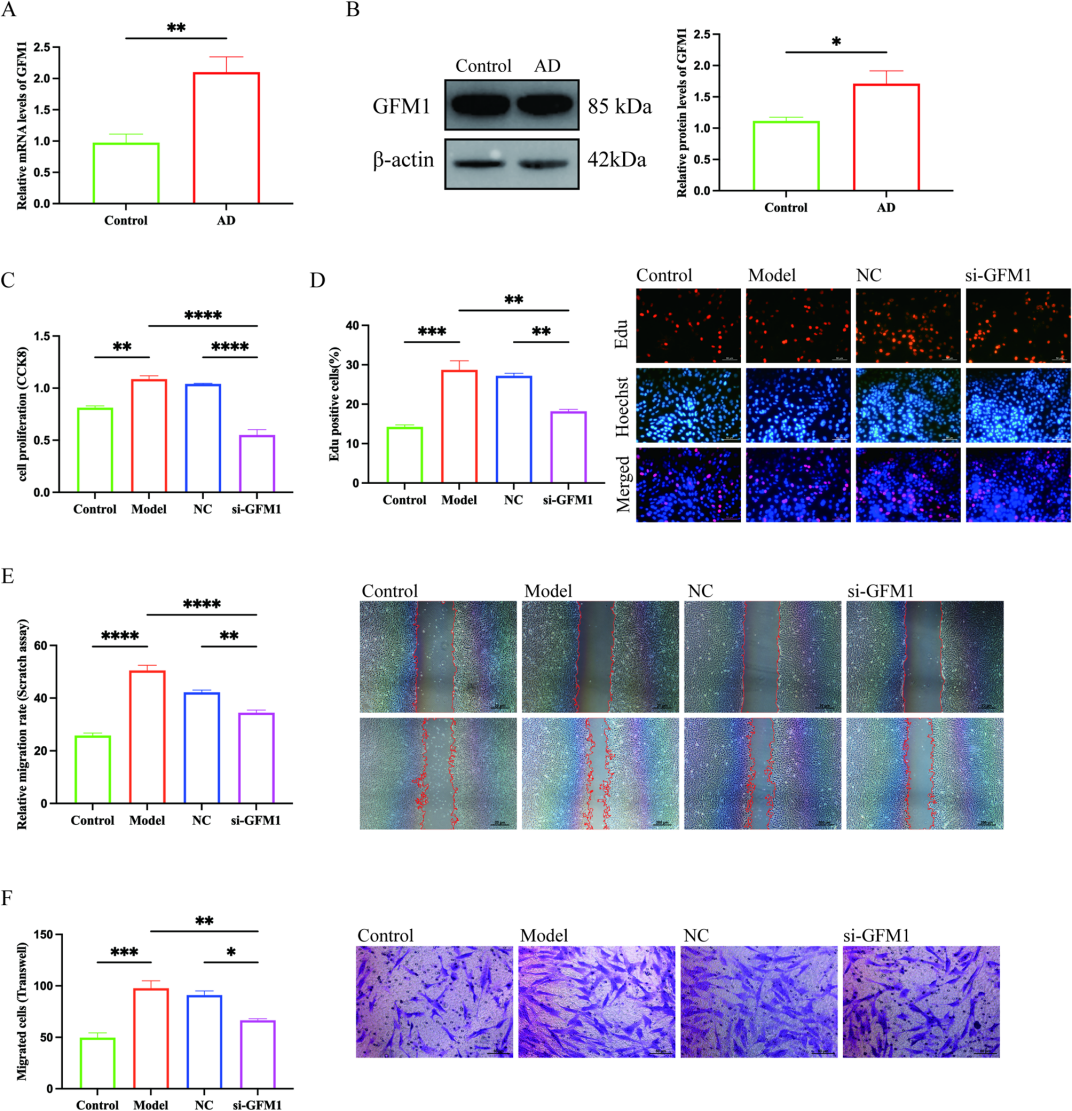
Validation of GFM1 expression and functional assays in mouse aortic VSMCs. (**A**) Relative GFM1 mRNA expression in human aortic tissues determined by qRT-PCR (β-actin as the reference gene). (**B**) Western blot and densitometric quantification of GFM1 protein in human aortic tissues (β-actin as the loading control). (**C**) CCK-8 assay (24 h) under four conditions: Control (untreated), Model (AngII 1.0 μmol/L for 24 h), NC (siNC + AngII), and siGFM1 (siGFM1 + AngII). (**D**) EdU incorporation assay (2 h incubation) with nuclear counterstain (blue) and quantification of EdU-positive cells. (**E**) Wound-healing assay at 0 h and 12 h with quantified migration/closure. (**F**) Transwell migration assay (24 h) with crystal violet staining and quantification of migrated cells. Data are presented as mean ± SD. Two-group comparisons were performed using an unpaired two-tailed Student’s t-test or Mann–Whitney U test, as appropriate. Comparisons among ≥3 groups were performed using one-way ANOVA with Tukey’s post hoc test or Kruskal–Wallis test with Dunn’s multiple-comparison test, as appropriate. P < 0.05 was considered statistically significant. Scale bars: 50 μm (EdU, Transwell); 200 μm (wound-healing).

In human aortic tissues, GFM1 mRNA and protein levels were higher in AD than in non-diseased aorta, as assessed by qRT-PCR and western blotting with densitometric normalization (Fig. 6A–B; group sizes are indicated in the figure). In cultured murine aortic VSMCs, angiotensin II (AngII) stimulation was associated with increased proliferation/viability and migration, reflected by higher CCK-8 and EdU readouts as well as increased Transwell migration and wound closure (Fig. 6C–F). siRNA-mediated GFM1 knockdown attenuated these AngII-associated responses across assays (Fig. 6C–F). Collectively, the tissue and cellular data support an association between elevated GFM1 expression and AD, and suggest that GFM1 may contribute to AngII-associated VSMC proliferative and migratory behavior. Statistical comparisons and exact P values are provided in Fig. 6.

## Discussion

AD is a life-threatening cardiovascular emergency for which effective strategies for prevention and treatment remain limited^1,5^. Despite progress in understanding its pathophysiology, clinical management is still constrained by the lack of widely validated biomarkers and actionable therapeutic targets^26^. Addressing these gaps will require molecular insights that enable earlier diagnosis and targeted intervention. Metabolic dysregulation has been reported in AD, but lactate-associated epigenetic regulation remains insufficiently defined and was not directly quantified in the present work. Here, we integrated public transcriptomic data with complementary machine-learning models (LASSO, random forest, XGBoost) as a prioritization strategy to nominate lactylation-related candidates in AD, and consistently prioritized GFM1 for preliminary validation.

Lactate is increasingly recognized as a signaling metabolite that can modulate gene expression through lactate-associated epigenetic programs, including histone lactylation^10^. In AD, VSMC dysfunction and extracellular matrix remodeling contribute to wall vulnerability, and metabolic shifts in VSMCs may increase lactate and be accompanied by changes in histone lactylation marks (e.g., H4K12la) and inflammatory transcriptional programs^27,28^. Consistent with this broader vascular literature, enhanced glycolysis supports inflammatory phenotypes in plaque immune cells^29,30^, and VSMCs can adopt glycolytic states that promote proliferation and migration^31^. TRAP1-related metabolic reprogramming has also been linked to lactate accumulation, H4K12la, and cellular senescence^32^. Within this context, we identified eleven lactylation-related DEGs enriched for glucose handling, mitochondrial function, and oxidative stress. However, lactylation itself was not directly quantified in AD tissues or in our VSMC experiments, and we did not determine whether GFM1 undergoes lysine lactylation; therefore, our findings should be interpreted cautiously as an association within a lactylation-related transcriptomic framework rather than direct evidence of GFM1 lactylation.

In contrast to studies that emphasize model development for prediction, we used machine-learning methods as supportive tools to prioritize biologically meaningful targets rather than to build a clinically deployable diagnostic model^33^. This integrative, biology-first strategy was designed to enhance the stability of candidate nomination while avoiding overinterpretation of internal performance metrics. In particular, cross-validated/hold-out AUC values were used only to summarize internal discrimination and were not intended to claim external predictive generalizability. Beyond discovery analyses, orthogonal validation by qPCR and western blotting confirmed concordant upregulation of GFM1 in AD. Experimental perturbation of GFM1 in VSMCs yielded reproducible effects on proliferation and migration, providing biological support for the computational prioritization and motivating targeted mechanistic investigation.

GFM1 encodes mitochondrial translation elongation factor G1, which is essential for mitochondrial protein synthesis and respiratory-chain integrity; pathogenic variants have been associated with combined oxidative phosphorylation deficiency in humans^34^. Although GFM1 has not been directly characterized in AD, mitochondrial dysfunction and metabolic remodeling are increasingly implicated in VSMCs phenotypic switching and aortic wall remodeling. AngII can promote mitochondrial ROS generation and redox signaling in vascular cells^35,36^, and mitochondrial homeostasis programs (e.g., PGC-1α-related pathways and SIRT3) have been linked to AngII-associated oxidative stress and aortopathy-related phenotypes^37,38^. In addition, pro-glycolytic signaling such as HIF-1α in VSMCs has been linked to AngII-driven vascular remodeling, and recent work supports a role for VSMCs metabolic reprogramming in AD pathogenesis^39,40^. Within this framework, GFM1 may represent a mitochondrial node that influences respiratory efficiency and redox tone, which could secondarily favor glycolytic adaptation and lactate accumulation under stress. This provides a rationale for why a mitochondrial factor emerged as a cross-model consensus candidate in a lactylation-related transcriptomic analysis. However, we did not directly measure mitochondrial function, metabolic flux, or lactylation; thus, these links should be regarded as testable hypotheses. Future work will combine GFM1 perturbation with mitochondrial functional assays and direct lactylation measurements, and will validate robustness in independent cohorts.

Several limitations should be acknowledged. First, the integrated public cohorts differ in sampling protocols, platforms, and clinical characteristics; residual heterogeneity and unmeasured confounding may influence gene prioritization and effect estimates. Second, tissue validation was performed in a single center with a modest sample size, and independent external validation is still required to establish generalizability and clinical value. Third, functional experiments were limited to an AngII-stimulated murine VSMC model and may not fully recapitulate human AD biology or contributions of other cell types. Fourth, cross-validation/hold-out AUC summarizes internal discrimination only and does not establish external predictive performance. Most importantly, we did not directly quantify protein or histone lactylation in AD tissues or in GFM1-silenced VSMCs; therefore, our findings should be interpreted as association within a lactylation-related transcriptomic framework rather than mechanistic proof at the lactylation level. Future work will prioritize external validation and direct lactylation profiling in tissues and relevant cellular models, together with in vivo and spatial/single-cell approaches to clarify cell-type–specific mechanisms.

## Conclusions

This integrative transcriptomic and machine-learning framework nominates GFM1 as a lactylation-related candidate associated with aortic dissection. The current evidence is associative and preliminary: we did not directly quantify protein or histone lactylation, and causality has not been established. Therefore, independent external validation in well-characterized cohorts, together with direct lactylation profiling and targeted mechanistic studies, will be necessary to confirm robustness and to clarify biological and potential translational relevance.

## Supplementary Information

Below is the link to the electronic supplementary material.

Supplementary Material 1

Supplementary Material 1

## Data Availability

The integrated transcriptomic data are available from GEO (GSE52093, GSE98770, GSE153434, GSE147026). Full uncropped gels and blots have been provided as Supplementary File 1. De-identified raw data underlying qPCR/WB and cell-based assays are available from the corresponding author upon reasonable request under an institutional agreement.
