# Supplementary material for: Machine-learning–guided transcriptomic integration identifies GFM1 as a lactylation-related candidate biomarker in aortic dissection
Source: Sci Rep. 2026 Feb 14;16:9033. doi: 10.1038/s41598-026-40139-9 (PMC12992784; doi:10.1038/s41598-026-40139-9)
Supplement: Supplementary file 1 — Supplementary Material 1 [file 41598_2026_40139_MOESM1_ESM.docx]

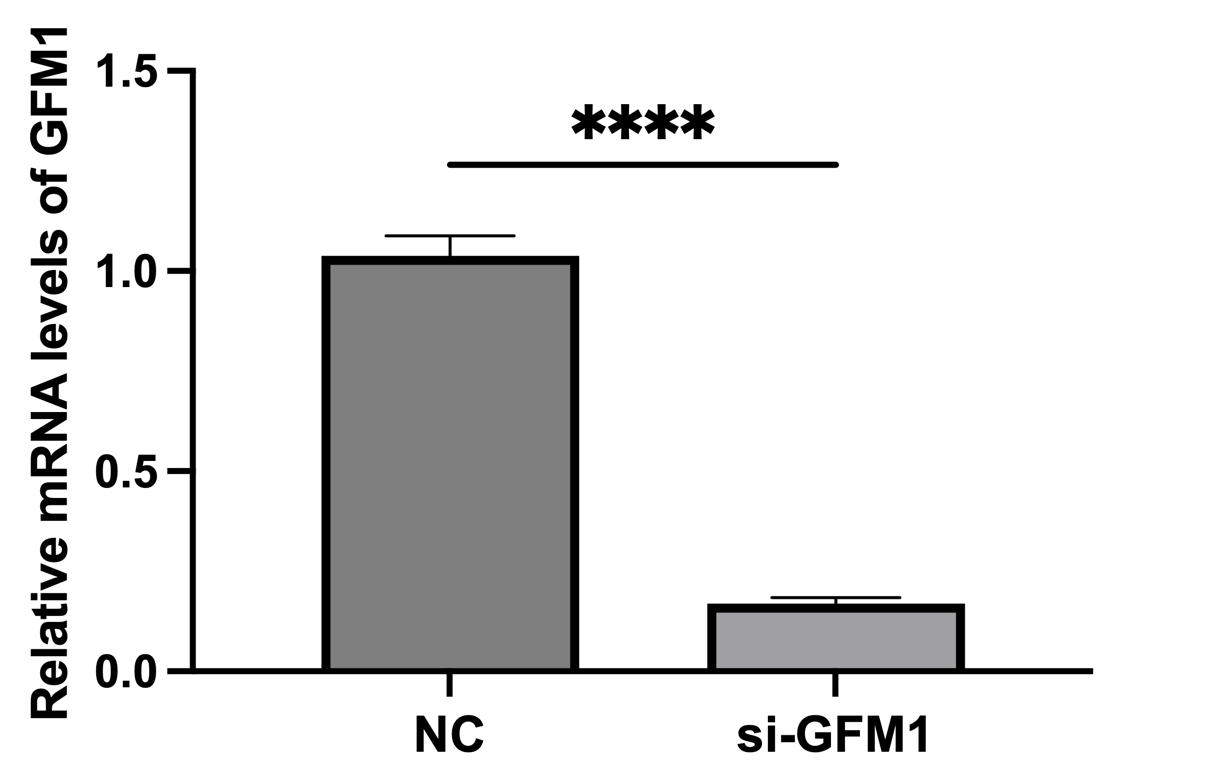


Supplementary Figure S1. qRT-PCR analysis of GFM1 knockdown efficiency in VSMCs 24 h after siRNA transfection (50 nM). Expression levels were normalized to β-actin and are shown as mean ± SD (n = 3 independent experiments). Statistical significance was assessed using an unpaired two-tailed Student’s t-test. P < 0.05 was considered statistically significant.
