# Supplementary figures and images for: Machine-learning–guided transcriptomic integration identifies GFM1 as a lactylation-related candidate biomarker in aortic dissection
Source: Sci Rep. 2026 Feb 14;16:9033. doi: 10.1038/s41598-026-40139-9 (PMC12992784; doi:10.1038/s41598-026-40139-9)

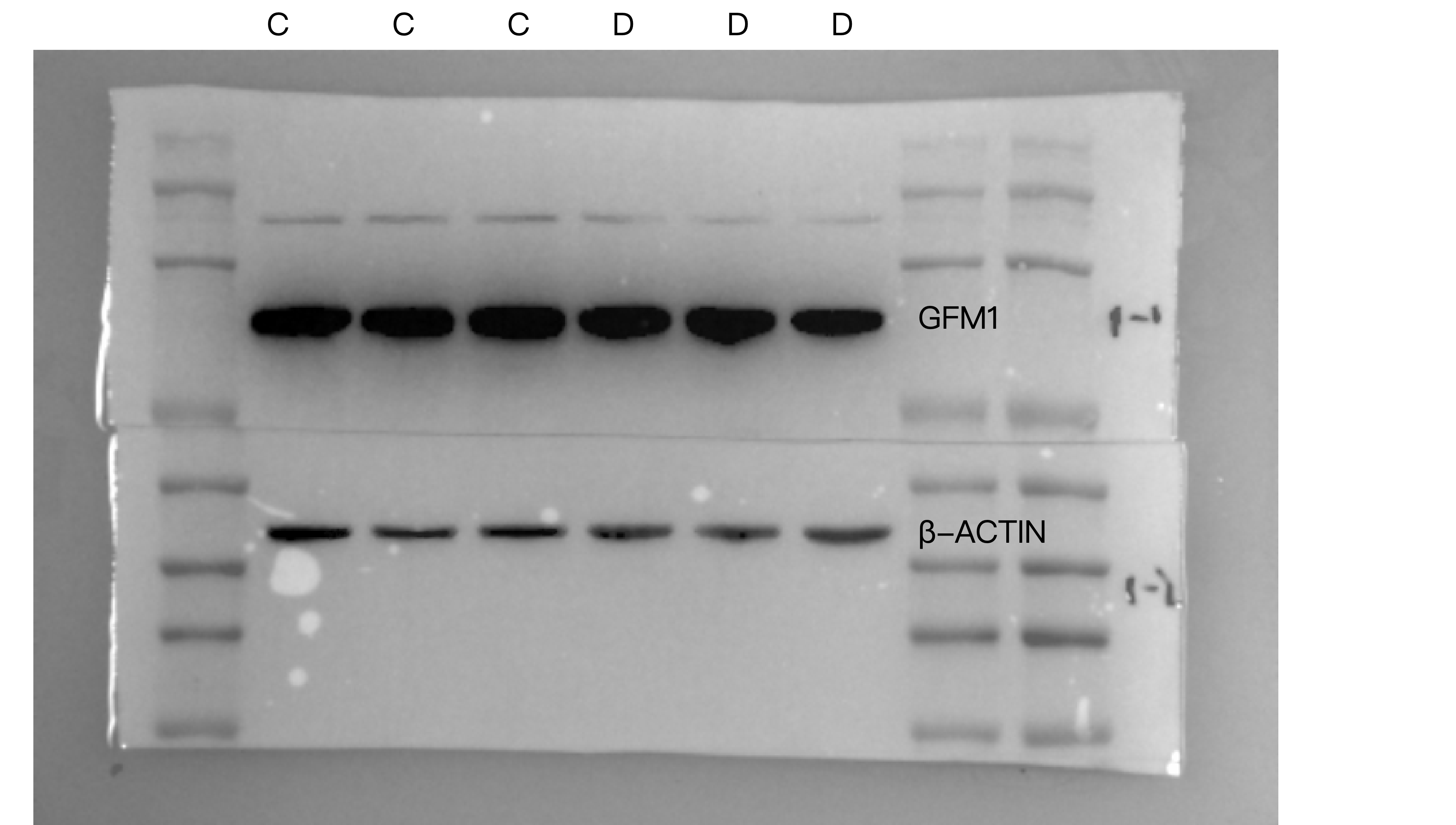

Supplement: Supplementary file 2 — Supplementary Material 1 [file 41598_2026_40139_MOESM2_ESM.png]
